# Lipoic Acid and Progesterone Alone or in Combination Ameliorate Retinal Degeneration in an Experimental Model of Hereditary Retinal Degeneration

**DOI:** 10.3389/fphar.2018.00469

**Published:** 2018-05-09

**Authors:** Dolores T. Ramírez-Lamelas, Soledad Benlloch-Navarro, Rosa López-Pedrajas, Roberto Gimeno-Hernández, Teresa Olivar, Dolores Silvestre, María Miranda

**Affiliations:** ^1^Departamento Ciencias Biomédicas, Universidad CEU Cardenal Herrera, CEU Universities, Valencia, Spain; ^2^Instituto de Ciencias Biomédicas, Universidad CEU Cardenal Herrera, CEU Universities, Valencia, Spain; ^3^Departamento Farmacia, Universidad CEU Cardenal Herrera, CEU Universities, Valencia, Spain

**Keywords:** retinitis pigmentosa, lipoic acid, progesterone, gliosis, glutathione

## Abstract

Retinitis pigmentosa (RP) is a group of inherited retinopathies characterized by photoreceptors death. Our group has shown the positive progesterone (P4) actions on cell death progression in an experimental model of RP. In an effort to enhance the beneficial effects of P4, the aim of this study was to combine P4 treatment with an antioxidant [lipoic acid (LA)] in the rd1 mice. rd1 and control mice were treated with 100 mg/kg body weight of P4, LA, or a combination of both on postnatal day 7 (PN7), 9, and 11, and were sacrificed at PN11. The administration of LA and/or P4 diminishes cell death in rd1 retinas. The effect obtained after the combined administration of LA and P4 is higher than the one obtained with LA or P4 alone. The three treatments decreased GFAP staining, however, in the far peripheral retina, and the two treatments that offered better results were LA and LA plus P4. LA or LA plus P4 increased retinal glutathione (GSH) concentration in the rd1 mice. Although LA and P4 are able to protect photoreceptors from death in rd1 mice retinas, a better effectiveness is achieved when administering LA and P4 at the same time.

## Introduction

Retinitis pigmentosa (RP) is a group of inherited retinopathies characterized by progressive photoreceptor death (Farrar et al., 2002; Daiger et al., 2013). In RP, rods typically die via mechanisms related to mutations, and afterward, cones degenerate more slowly secondarily in a mutation-independent manner (Pierce, 2001). RP accounts for approximately half of the cases of hereditary retinal diseases worldwide (Daiger et al., 2013).

Although today, more than 100 genes have been linked to RP, the exact mechanisms underlying this retinal degeneration that lead to photoreceptor death remain controversial. Indeed, although some retinal changes result as a direct mutation effect, some other changes result from modifications of the transcriptional system (Sancho-Pelluz et al., 2008). A substantial body of data suggests that increase oxidative stress, alterations in the inflammatory response, and retinal remodeling also play a crucial role in RP pathogenesis (Sanz et al., 2007; Noailles et al., 2014; Jones et al., 2016; Sánchez-Vallejo et al., 2016).

The development of genetic treatments for diseases with enormous genetic diversity, such as RP, implies problems because the number of patients with the same mutation is very small. This situation means that gene therapies need to be tailored for each patient. A good therapeutic strategy could be to attack common downstream pathways that lead to photoreceptor degeneration, as this could provide benefits to most RP patients (Sancho-Pelluz et al., 2008; Wang et al., 2014). In this sense, although various approaches have been explored to delay photoreceptor cells in RP, including the administration of antioxidants and other neuroprotective agents, no effective treatment is currently available for RP patients (Wang et al., 2014). Contributing to the therapeutic limitations of these treatments might be the fact that they target a single aspect or mechanism of the disease.

To develop effective treatments, we postulate the use of multifunctional agents or a combination of different substances that would be able to affect several factors influencing disease progression, such as oxidative stress and inflammation, which have a key role in RP.

Our group has recently shown the positive actions of progesterone (P4), a neurosteroid hormone, on the progression of cell death in the rd1 mice, an RP experimental model (Sánchez-Vallejo et al., 2015). Baulieu et al. (1996) coined the term neurosteroids to refer to those steroids that may be synthesized in the central nervous system (CNS) and affect neurotransmission. The neurosteroids’ neuroprotective actions are dependent more on their chemical structure than on their properties as hormones (Moosmann and Behl, 1999). In this sense, it has been shown that P4 is a worse antioxidant than estradiol and other estrogens (Mooradian, 1993). In an effort to enhance the beneficial effects of P4, the aims of this study were to combine P4 treatment with a widely known and effective antioxidant [α-lipoic acid (LA)], in order to increase the antioxidant properties of our treatment, and to establish the changes induced by this combined treatment in the retina of rd1 mice.

P4 is widely known for its role in sexual reproduction, but during the last decade, increasing evidence of the neuroprotective role of neurosteroids has been obtained. P4 has been successfully used in different clinical trials for the treatment of traumatic brain injury (Wright et al., 2007). These P4 neuroprotective effects may be due to its action on numerous processes and signaling pathways, such as the decreased release of inflammatory cytokines, decreased cellular apoptosis, positive regulation of the γ-aminobutyric acid neurotransmitter (GABA), and decreased lipid peroxidation and oxidative stress (Stein, 2008).

The potential use of P4 in some experimental retinal degenerations, such as retinal ischemia and in animal models of RP has been demonstrated (Allen et al., 2015; Sánchez-Vallejo et al., 2015). Recently, other studies have shown that the administration of norgestrel, a synthetic progestin, protects photoreceptors in models of retinal light damage in mice or in a model of retinal degeneration (the rd10 mice) (Doonan et al., 2011; Doonan and Cotter, 2012).

LA is a natural molecule with antioxidant and anti-inflammatory properties. It can exert indirect antioxidant actions, such as the induction of glutamate cysteine ligase (GCL), the key-limiting enzyme in glutathione (GSH) synthesis, and other phase II enzymes (Ji et al., 2013). LA can also be converted to dihydrolipoic acid, which can regenerate several antioxidants, such as vitamin C, GSH, and α-tocopherol (Packer et al., 1997). It can modify mitochondrial calcium transport (Schweizer and Richter, 1996) and can act as a modulator of different transduction signaling pathways, such as nuclear factor kappa B (Suzuki et al., 1992; Golbidi et al., 2011). All these actions may explain the beneficial effects of LA observed in different neurodegenerative disorders.

LA has also been shown to be useful in ocular diseases, such as glaucoma, diabetic retinopathy, dry eye, and cataracts (Kowluru and Odenbach, 2004; Johnsen-Soriano et al., 2008; Inman et al., 2013; Andrade et al., 2014; Pescosolido et al., 2016). There is also evidence that LA (alone or with other antioxidants) is able to decrease retinal oxidative stress in mouse models of RP (Sanz et al., 2007; Zhao et al., 2014).

In this study, we sought to study the potential synergistic effects of LA and P4 in the delay of photoreceptor death in rd1 mice, and to observe their effects on several markers of gliosis and antioxidant defenses, two of the mechanisms that have been related to PR pathogenesis.

## Materials and Methods

### Ethics Statement

Animal care and other protocols followed the Association for Research in Vision and Ophthalmology statement for the Use of Animals in Ophthalmic and Vision Research. Experiments were authorized by the Animal Ethics Committee of CEU Cardenal Herrera University (reference 11/013).

### Animal Care

Male and female control and rd1 mice were housed in cages under light-dark cycles of 12 h. During housing, the animals had *ad libitum* free access to water and to a diet for small laboratory animals (Harlan Ibérica S.L., Barcelona, Spain).

The day of mice birth was considered as PN 0 (post-natal day 0). In general, previous studies found that the degeneration of rods in the retina of rd1 mice occurs drastically at PN 11 during the morphogenesis of the photoreceptors. Therefore, comparisons in this study among these animals and the control mice were carried out at PN 11. Mice were decapitated after asphyxiation in a CO_2_ chamber.

### Experimental Design

rd1 and control mice (C3H) were used to assay the potential LA (Sigma-Aldrich, Madrid, Spain) and/or P4 (Sigma-Aldrich, Madrid, Spain) protective action. rd1 mice were a gift from Dr. Van Veen from Lund University. The rd1 mouse was first discovered by Keeler (Keeler, 1924) and is characterized by a mutation in the gene encoding for the β-subunit of rod photoreceptor cGMP phosphodiesterase 6 (PDE6) (Bowes et al., 1990) which leads to the accumulation of cGMP in rod photoreceptors (Farber and Lolley, 1974). The degenerative process in this model starts very early and the peak of rod photoreceptor cell death occurs between PN 11-14 (Punzo and Cepko, 2007). Mutations in the PDE6-β gene are also present in about 10% of RP human patients (Bayés et al., 1995).

The mice were divided into the following four groups: group 1, which consisted of control and rd1 mice that were not subjected to any treatment to be used as control, but were administered a corresponding dose of vehicle (olive oil); group 2, which consisted of control and rd1 mice that were orally administered 100 mg/kg body weight of LA diluted in olive oil; group 3, which was composed of control and rd1 mice that were given an oral administration of 100 mg/kg body weight of P4 diluted in olive oil; and group 4, which consisted of control and rd1 mice that were given a combined administration of LA (100 mg/kg body weight) and P4 (100 mg/kg body weight). A stock solution of P4 (20 mg/ml) or LA (20 mg/ml) in olive oil was prepared at the beginning of the experiment. The amount of solution administered to each animal depended on the body weight (approximately 20–30 μl). Mice received this solution with the help of a gastric tube. Vehicle or treatments were administered to the mice on alternate days starting at PN 7; the last day of treatment was PN 11. The animals received 3 doses of LA, P4, or LA+P4 or vehicle (at PN7, PN9, and finally at PN11). It has been demonstrated that LA and P4 have a rapid gastrointestinal absorption (reaching serum peak concentrations after 1 h for LA and 3 h for P4) as well as a rapid clearance from the body (Maxson and Hargrove, 1985; Brufani and Figliola, 2014). For this reason, the animals were sacrificed 8 h after the administration of the last treatment dose. The dose of LA and P4 used in this study was the same as those used by our group in previous studies (Sánchez-Vallejo et al., 2015; Paradells-Navarro et al., 2017). The dose-ranging effect has been studied comparing the results obtained in this study with the ones obtained previously by other group (Miranda et al., 2010; Sánchez-Vallejo et al., 2015). In addition, a dose-effect curve was also performed for P4 in another animal model of RP, the rd10 mouse that has a mutation in the same gene that the rd1 mouse. Different doses of P4 (50, 100, 150, and 200 mg/kg body weight) have been administered orally to the mice and the number of rows of photoreceptors in the outer nuclear layer (ONL) of the retina was quantified.

### Histologic Processing

After the mice were sacrificed, the eyes were dissected and fixed in 4% paraformaldehyde solution for 2 h. After three washes with 0.1 M phosphate-buffered saline (PBS) pH 7, eyes were cryoprotected using increasing concentrations of PBS sucrose. Retinal 8 μm sections were made in a Leica CM 1850 UV AgProtect cryostat (Leica Microsystems SLU, Barcelona, Spain) on Menzel-Gläser Super Frost^®^ Plus ports (Thermo Fisher Scientific, Braunschweig, Germany), and then they were stored at -20°C until use. These retinal cryosections were used to study retinal dying cells with terminal deoxynucleotidyl transferase dUTP nick end labelling (TUNEL) technique and to examine glial fibrilary acidic protein (GFAP) and GCL catalytic subunit (GCLC). In rd1 mice, photoreceptor death takes place differently depending on the area of the retina; therefore, we divided the retina into the following three sections to express our results: far peripheral, middle peripheral, and central retina.

TUNEL assay was performed with an *in situ* cell death detection kit (Roche Diagnostics, Mannheim, Germany) in accordance to the manufacturer’s instructions. TUNEL positive cells were counted in the three retinal areas specified before at 20× amplification, and the result was divided by the value of the ONL area. All data are presented as the means and standard errors from three retinal sections for six different animals.

Immunohistochemistry was performed for the detection of GFAP and GCLC. Sections were dried at room temperature (RT). Then, the tissue was rehydrated and blocked for 1 h at RT with 20% of normal goat serum in PBS. Sections were incubated overnight, all at 4°C, with primary antibodies [GFAP, 1:500 (Dako, Denmark GSH); GCLC, 1:100 (Abcam, Cambridge, United Kingdom)] diluted in PBS-BSA-Triton 0.3%. Sections were washed three times and incubated for 60 min in the dark at RT with secondary antibodies (Alexa 488 goat anti-rabbit; Invitrogen, Life Technologies, Madrid, Spain). The sections were rinsed again, and mounted using Vectashield medium (Burlingame, CA, United States).

Fluorescence microscopy was performed with a Leica DM2000 microscope with a Nikon DS-Fi1 camera using the Leica application suite version 2.7.0 R1 software (Leica Microsystems, Barcelona, Spain). Image processing was realized with the help of Adobe Photoshop CS5. The percentage of area dyed was quantified in GFAP and GCLC immunostainings by using Image J 1.45s.

### Glutathione Assay

Retinal homogenates were prepared as described in Sánchez-Vallejo et al. (2015) and reduced and oxidized GSH (Glutathione-S-S-Glutathione; GSSG) concentrations in these homogenates were quantified by the method of Reed et al. (1980) as described by Sánchez-Vallejo et al. (2015). Protein concentration was determined by means of the Lowry method (Lowry et al., 1951).

### Statistical Analysis

Numerical data are expressed as mean ± standard error of the mean (SEM). Kolmogorov–Smirnov test was performed to confirm the normality of our data distribution, followed by Levene’s test to estimate variance homogeneity (*p* > 0.01). Data were statistically analyzed using one-way ANOVA and Fisher’s LSD or Games–Howell *post hoc* analysis was carried out (differences were considered significant if *p* < 0.05).

## Results

### LA and/or P4 Decreased Photoreceptor Cell Death in rd1 Mice

**FIGURE 1:**
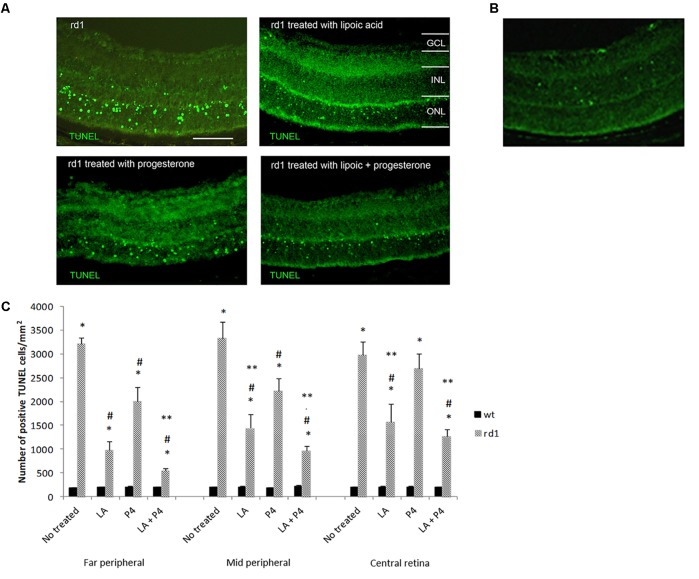
Evaluation of TUNEL staining in non-treated and treated control and rd1 retinas. **(A)** Fluorescence TUNEL cells in central retinas from non-treated, treated with lipoic acid, treated with progesterone and treated with lipoic acid and progesterone rd1 mice. (Scale bar: 50 μm). **(B)** Fluorescence TUNEL image in control mice central retina at PN11. **(C)** Quantification of the number of TUNEL positive cells at PN11 [postive cells/arbitrary units of area (AUA)] in the retina from the animal groups examined (treated and non-treated control and rd1 mice) in three retinal regions (6 animals for strain and treatment) (^∗^*p* < 0.02 vs. control; ^#^*p* < 0.03 vs. non treated rd1 mice; ^∗∗^*p* < 0.02 vs. P4 treated rd1 mice).

Either LA or P4 reduced the number of TUNEL-positive photoreceptors in the ONL of the rd1 retina (**Figure 1A**). **Figure 1B** shows a fluorescence TUNEL image in control mice central retina at PN11. **Figure 1C** indicates the number of total positive cells for TUNEL assay in the three retinal areas considered (far peripheral, middle peripheral, and central retina) in the control and rd1 mice non treated or treated with LA, P4, or LA plus P4. All groups of rd1 mice (non-treated and treated ones) showed an increment in the number of TUNEL-positive cells when compared with their respective control groups (^∗^*p* < 0.02 vs. control groups). However, the administration of either LA or P4 and the combination of LA plus P4 diminish cell death in rd1 mouse retinas (^#^*p* < 0.03 vs. non-treated rd1 mice), except for P4, which could not decrease photoreceptor death in the central retina. These results confirm that both substances have neuroprotective properties. When comparing the effectiveness of these two molecules, we observed that the effect of LA or LA combined with P4 is higher than the one observed only with P4, mainly in the mid-peripheral and central retina (^∗∗^*p* < 0.02 vs. P4 rd1-treated mice). It is important to highlight that, in the far and mid peripheral retina, the effect obtained after the combined administration of LA and P4 is higher than the one obtained with LA or P4 alone.

To better characterize this possible synergistic effect, we have quantified the percentage of decrease in the number of retinal TUNEL cells (**Table 1**). We can observe that the greatest reduction in the number of TUNEL-positive cells occurs in the far peripheral zone, with a diminution of 40% in those rd1 animals given only P4, a 70% reduction in the percentage of TUNEL-positive cells in those mice that were administered LA, and approximately 80% decrease in those rd1 mice who received a combined dose of LA and P4. In middle peripheral and central retina there was also a greater reduction of TUNEL-positive cells when LA and P4 were administered at the same time than when P4 was administered alone, though these treatments were not as effective as in the far peripheral retina.

**Table 1:** Percentage of decrease positive cells for TUNEL quantification in the retina of mice treated with LA and/or P4 in the three retinal sections studied, considering 100% the number of TUNEL-positive cells in non-treated rd1 mice (^∗^*p* < 0.05 vs. LA+P4 rd1 mice; ^#^*p* < 0.05 vs. LA treated mice).

	Far peripheral retina	Middle peripheral retina	Central retina
% decrease in LA-treated mice	69.40	56.65	9.50
% decrease in P4-treated mice	37.33^∗#^	33.51^∗^	47.33^∗^
% decrease in LA+P4-treated mice	82.78	71.10	57.30


The results obtained in this study are closely related to the dose of LA and P4 used. In a previous work (Miranda et al., 2010), administration of LA at a dose of 10 mg/Kg body weight during 8 days (from PN3 to PN10) resulted in only a reduction of 4.57% of the TUNEL-positive cells in the retina of the rd1 mice. Herein, we describe that the administration of 100 mg/kg body weight of LA during 3 days was able to reduce 45.18% (average) of the TUNEL-positive cells in the ONL of these animals. In addition, a dose of 100 mg/kg of LA has also shown to be beneficial in a brain injury model (Paradells-Navarro et al., 2017). Regarding the administration of oral P4 to animals with retinal degenerations, the dose of 100 mg/kg has shown to be effective in protecting photoreceptors in rd1 mice (Sánchez-Vallejo et al., 2015). In addition, we have compared the effect of different doses of P4 (50, 100, 150, and 200 mg/kg body weight) in another retinal degeneration mice and we have demonstrated that the first dose that was able to show a significant improvement in photoreceptor death was 100 mg/kg body weight (Supplementary Figure S1).

### Characteristic Gliosis Observed in the Retina of the rd1 Mice Is Decreased After Oral Treatment With LA, P4, and Their Combination

**FIGURE 2:**
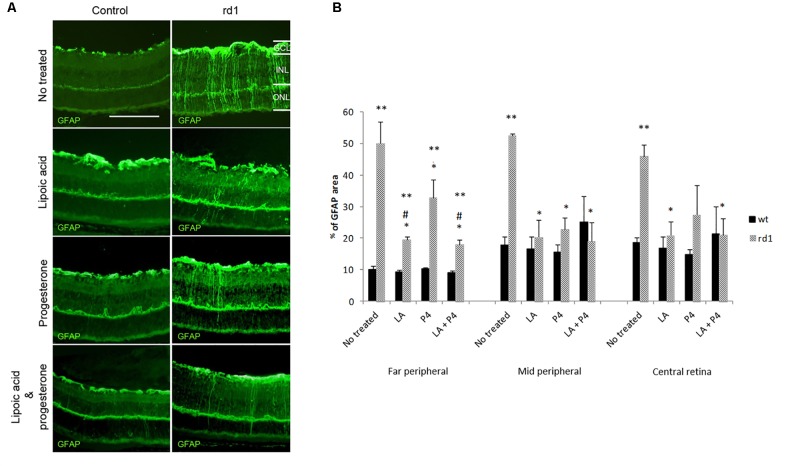
GFAP retinal immunostaining. **(A)** Examples of images of fluorescent labeling for GFAP in central retinas from non-treated, treated with LA, treated with progesterone and treated with LA and progesterone rd1 mice (images on the left corresponds to control mice and images on the right corresponds to rd1 mice) (Scale bar: 50 μm). **(B)** Histogram comparing GFAP (% stained area) in retinas non-treated, treated with LA, treated with progesterone and treated with LA and progesterone rd1 mice (three animals for strain and treatment; ^∗∗^*p* < 0.02 vs. control mice, ^∗^*p* < 0.02 vs. non-treated rd1; ^#^*p* < 0.05 vs. P4 treated rd1 mice).

In healthy retinas, GFAP expression is mainly localized in astrocytes. However, the overexpression of GFAP can be used as an indicator of stress and retinal damage, as well as the activation of Müller cells (Luna et al., 2010). **Figure 2A** shows the positivity towards the GFAP protein of Müller cells that become reactive during the degenerative process in rd1 mice when compared with control retinas.

In this study, GFAP immunoreactivity was quantified measuring the ratio of the total area in each part of the retina that was stained with the secondary antibody (**Figure 2B**). Our results confirmed the characteristic retinal gliosis observed in non-treated rd1 mice in the three retinal areas studied compared with the control mice (^∗∗^*p* < 0.02 vs. control). Though the three different oral treatments administered in this study to rd1 mice (LA, P4, or LA plus P4) were able to decrease GFAP staining in Müller cells (^∗^*p* < 0.02 vs. rd1 not treated), the effect of these treatments was different according to the part of the retina studied. LA and P4, as well as the combination of LA and P4, showed similar effects in the middle peripheral. However, in the far peripheral and central retina, the effectiveness of the treatments was less remarkable, and they could not completely reverse Müller cells’ activation. Interestingly, in the far peripheral retina, the treatment that offered better results was LA plus P4 (^#^*p* < 0.05 vs. P4-treated mice). This is the first time that this anti-gliosis mechanism is described for LA and LA+P4 in this animal model.

### Expression of Glutamate Cysteine Ligase Catalytic Subunit Enzyme in Control and rd1 Mice Retina

**FIGURE 3:**
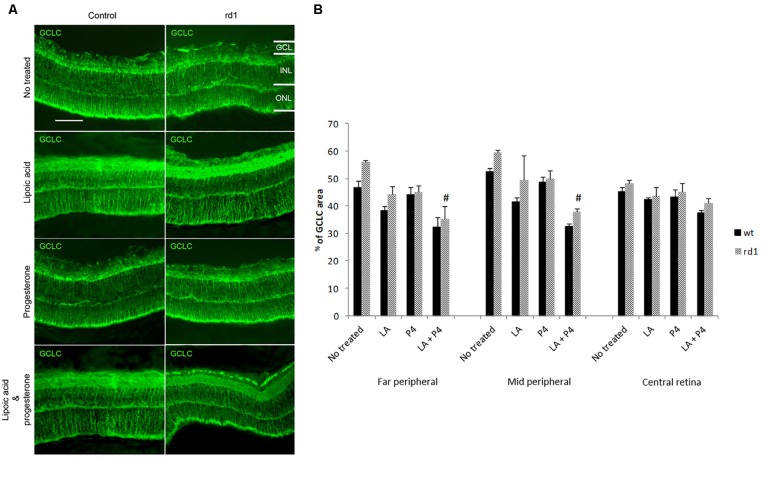
Retinal GCLC. **(A)** Central retina immunolabelled for GSH from non-treated, treated with LA, treated with progesterone, and treated with LA and progesterone rd1 mice (images on the left correspond to control mice and images on the right correspond to rd1 mice; scale bar: 50 μm) **(B)** Quantification of GCLC (percentage of stained area) in retinas from non-treated, treated with LA, treated with progesterone, and treated with LA and progesterone rd1 mice (three animals for strain and treatment; ^#^*p* < 0.01 vs. non-treated rd1 mice).

Representative images of GCLC staining in the central retina of control (without and with treatment) and rd1 (without and with treatment) mice are shown in **Figure 3A**. GCLC immunoreactivity was quantified by measuring the ratio of the total area in each part of the retina that was stained with the secondary antibody (**Figure 3B**). No differences were observed between GCLC expression in the control and rd1 mice in the three retinal zones studied. Treatment with LA or P4 did not modify the expression of this enzyme; however, we observed a significant reduction in GCLC in the far and middle peripheral not treated rd1 retina and those treated with the combination of LA plus P4 (^#^*p* < 0.01).

### Lipoic Acid Increases GSH Retinal Concentration

**FIGURE 4:**
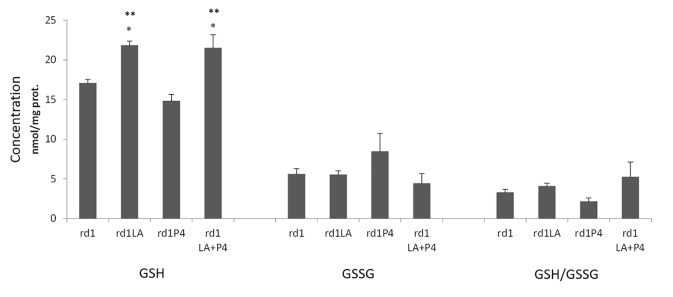
GSH and GSSG retinal concentrations and GSH/GSSH ratio (^∗^*p* < 0.01 vs. non treated rd1 mice; ^∗∗^*p* < 0.01 vs. P4 treated mice; 4 animals for strain and treatment).

LA or LA combined with P4 treatment significantly increased GSH retinal concentration in rd1 mice. However, no effect was observed in the GSSG concentration or the GSH/GSSG ratio (**Figure 4**). The treatment with P4 did not modify the GSH or GSSH concentration.

## Discussion

Our group and others have previously described the possible therapeutic actions of P4 and P4 analogues in different retinal degenerations animal models, such as the rd1 and the rd10 mice (Doonan et al., 2011; Sánchez-Vallejo et al., 2015). However, the effect on photoreceptor survival is only partial, and a complete prevention is not achieved. To enhance the effect of progesterone, in this study, we have administered P4 and an antioxidant. The antioxidant we have selected is LA, which has been proposed to be therapeutically useful in several neurodegenerative diseases (Irwin et al., 2016).

It is well known that both L4 and P4 are able to cross the blood–brain and blood–retinal barriers. After oral intake, LA is absorbed in the gastrointestinal system and is transported to different organs such as the brain (Gomes and Negrato, 2014). The LA beneficial effects on CNS diseases have been described widely (Seifar et al., 2017) and its oral tolerability in order to administer it for eye diseases has also been demonstrated (Sarezky et al., 2016). Regarding P4, its potential beneficial effect after traumatic brain injury has been demonstrated (Shahrokhi et al., 2010).

The involvement of neurosteroids in the physiology and pathology of visual function has been proven both in humans and in experimental animals (Guarneri et al., 2003).

In addition, progesterone receptors have been identified in photoreceptor and Müller cells (Swiatek-De Lange et al., 2007).

In this study, LA and P4 decreased the number of TUNEL-positive cells at ONL of PN11 rd1 retinas after being administered for three days (**Figure 1**). In fact, LA has a greater effect when compared with the P4 effect, mainly in the mid-peripheral and central retina. However, in the peripheral retina, the effect obtained after the combined administration of LA and P4 is higher than the one obtained with LA or P4 alone (**Figure 1**). This effect observed, mainly in the far peripheral retina, may be related to the fact that the degeneration in rd1 mice typically begins in the periphery and progresses to the center of the retina (Jones and Marc, 2005). This result allow us to confirm for the first time the utility of adding an antioxidant to a neurosteroid for retinal degenerations treatment. Increasing evidence demonstrates that steroid gonadal hormones not only play an important role on the reproductive system but also on other systems, such as the CNS. As it has been previously mentioned, the neuroprotective actions of neurosteroids are dependent more on their chemical structure as antioxidants than on their properties as hormones (Moosmann and Behl, 1999) and this is a possible explanation for the increased effectiveness of the combined retinal with LA and P4, compared to the results obtained only with P4 or LA, observed in this work.

Other authors have combined P4 with other antioxidants to improve its effectiveness in other experimental paradigms. In this sense, Atif et al. (2013) evaluated the neuroprotective effects of the combination of progesterone and 25-dihydroxyvitamin D on primary cortical neurons subjected to glucose deprivation in rats with ischemic injury. Their results revealed that this combination protected the brain better than P4 treatment.

More recently, it has been shown that norgestrel protection in the rd10 retina was attenuated in the presence of two different antioxidants (Ruiz-Lopez et al., 2017). The researcher hypothesis is that norgestrel upregulates the production of “pro-survival” reactive oxygen species (ROS) and that ROS may have a detrimental or a protective role, depending on different aspects within the cells (i.e., location, intensity, etc.; Ruiz-Lopez et al., 2017). These latter results may seem contradictory to the results from our study; however the antioxidants used were different (4,5-Dihydroxy-1,3-benzenedisulfonic acid disodium salt or *N*-acetyl-L-cysteine) from the one we have used (LA).

Retinal degenerations are usually accompanied by the reactive gliosis of Müller cells. Though it is not clear if this gliosis is associated with protective or detrimental effects, it is well known that, as retinal degeneration progresses, a glial scar can be formed in the entire retina and interfere with neuronal survival and regeneration (Hippert et al., 2015).

We have also studied if LA an P4 and their combination were able to decrease the typical gliosis observed in the rd1 retina. Treatment with LA and P4 not only alleviated retinal cell death but also simultaneously decreased the expression of the GFAP protein (**Figures 2A,B**). These results confirm and quantify our previous results regarding P4 (Sánchez-Vallejo et al., 2015) However, it is the first time that we can confirm that LA has also this anti-gliosis effect on the rd1 retina and that this effect is even higher than the one obtained with P4 (mainly in the far peripheral retina). In addition, the combined treatment of LA and P4 decreases gliosis to a greater extent that P4 treatment alone (**Figure 2B**), possibly due to the well known LA anti-inflammatory properties.

In other tissues, various studies have shown that LA works as a good neuroprotective agent by acting as an antioxidant and anti-inflammatory agent (Freitas, 2009; Toklu et al., 2009). The fundamental role of oxidative stress in the pathogenesis and progression of the inflammatory process is well known. In this sense, it is not easy to establish a separate effect on the inflammation or oxidative stress of most of the antioxidants usually used. Many other substances such as resveratrol, melatonin, vitamin A, lutein, zeaxanthin, etc. (Kubota et al., 2009; Bian et al., 2012; Tian et al., 2013; Petiz et al., 2017; Tuzcu et al., 2017) have shown antioxidant and anti-inflammatory properties in the retina and in other tissues. However, the relation between antioxidant therapy and inflammatory diseases needs more study.

Oxidative stress is not the direct mechanism of photoreceptor cell death in this RP animal model. However, it may play an indirect role as it has been previously shown that antioxidants may delay the progression of RP (Sanz et al., 2007). For this reason, we examined several markers of this process in control and rd1 mice: GCLC, GSH and GSSG (reduced and oxidized glutathione) retinal concentrations, and the ratio GSH/GSSG.

We have not observed any differences in the expression of GCLC in the retinas from control and rd1 mice at PN11, the peak of cell death in this mice model. These results agree with previous studies from our group in which only an increment in retinal GCLC in rd1 mice was observed after the maximum peak of cell death (PN19) (Sánchez-Vallejo et al., 2016).

It is interesting to note that a decrease in the expression of GCLC is observed in rd1 mice treated with the combination of LA and P4, and that this decrease is only observed in the far and middle peripheral retina. Herein, we postulate that the mice that received both treatments are the mice with a greater delay in photoreceptor death (**Table 1**). Therefore, they may be less exposed to oxidative stress and in such conditions it is not necessary to synthesize a new antioxidant defense. Somehow, this enzyme may be downregulated.

Glutathione is a peptide that defends against oxidative stress by scavenging free radicals and other reactive species (Dalazen et al., 2014). GSH is synthesized in the cytosol of all cells and involves two enzymatic steps. The first step is considered the limiting step and is catalyzed by γ-glutamylcysteine synthetase or GCL (Meister and Anderson, 1983). In addition, GSH exists in the form of reduced thiol (GSH), which predominates in the cell, and oxidized disulfide (GSSG) (Kaplowitz et al., 1985). Intracellular GSH can reduce H_2_O_2_ with the enzyme glutathione peroxidase (GPx). In this course of action, GSH is oxidized to GSSG, which is reduced again to GSH by the enzyme GSSG reductase (GR) (Ganea and Harding, 2006). In situations of severe oxidative stress, the ability of the cell to convert GSSG to GSH may be decreased, leading to GSSG accumulation (Lu, 1999).

We have shown that retinal GSH concentration is increased in those animals that were administered LA or LA and P4 (**Figure 4**). It is known that LA may increase GSH concentration because it may stimulate GCLC, or it may increase the conversion from GSSG to GSH (Paradells-Navarro et al., 2017). Nevertheless, we have not observed any changes in GCLC expression (**Figure 3**), GSSG concentration, or GSH/GSSG ratio (**Figure 4**). We may suggest two possible explanations to this GSH increase, that is not accompanied by any change in GCLC expression or GSSG concentration: (i) in mammalian cells, three mechanisms serve to maintain the GSH homeostasis, *de novo* synthesis, the catalyzed reduction of GSSG but also an extracellular uptake (retinal GSH may be increased because there is an uptake from other parts of the visual system) and (ii) it has been demonstrated that there is not a direct correlation between changes in GCLC expression and changes in GSH concentration and that, for example, the knockout of the mouse GCLC gene is responsible for only about 20% diminution in GSH levels (Dalton et al., 2000).

We can conclude that although LA, P4, and their combined oral treatment are able to protect photoreceptors from death in the retinas of rd1 mice, a better effectiveness is achieved when administering LA and P4 at the same time. *In vivo*, the characteristic gliosis detected in the retina of the rd1 mouse model is decreased and GSH retinal concentration is increased after oral treatment of LA and P4 at the same time. Our results also suggest that both LA and P4 may protect the retina through antioxidant and anti-inflammatory mechanisms, but a better effectiveness is achieved when both substances are administered together.

## Author Contributions

MM, DS, and TO designed the experiments. DR-L, SB-N, and RG-H performed the experiments. MM, RL-P, and DS wrote the paper. All authors reviewed the manuscript.

## Conflict of Interest Statement

The authors declare that the research was conducted in the absence of any commercial or financial relationships that could be construed as a potential conflict of interest.
