# Supplementary figures and images for: Lipoic Acid and Progesterone Alone or in Combination Ameliorate Retinal Degeneration in an Experimental Model of Hereditary Retinal Degeneration
Source: Front Pharmacol. 2018 May 9;9:469. doi: 10.3389/fphar.2018.00469 (PMC5954235; doi:10.3389/fphar.2018.00469)

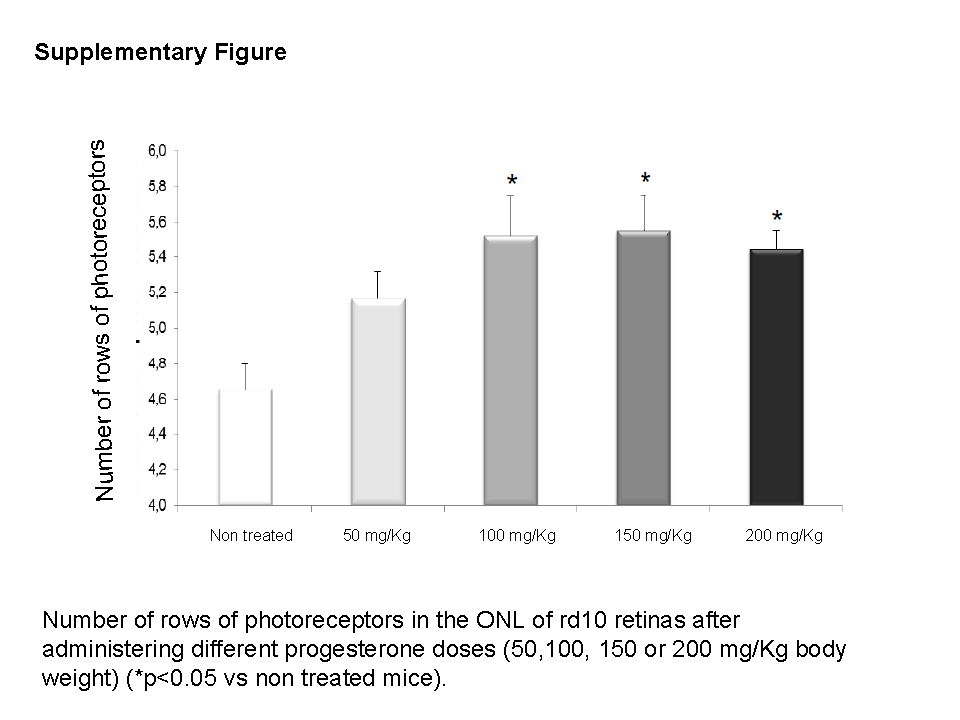

Supplement: Supplementary file 1 [file Image_1.tif]
